# Medium-Term Increases in Ambient Grass Pollen Between 1994-1999 and 2016-2020 in a Subtropical Climate Zone

**DOI:** 10.3389/falgy.2021.705313

**Published:** 2021-08-05

**Authors:** Beth Addison-Smith, Andelija Milic, Divya Dwarakanath, Marko Simunovic, Shanice Van Haeften, Victoria Timbrell, Janet M. Davies

**Affiliations:** ^1^School of Biomedical Sciences, Centre for Immunology and Infection Control, Centre for the Environment, Queensland University of Technology, Brisbane, QLD, Australia; ^2^Office of Research, Metro North Hospital and Health Service, Brisbane, QLD, Australia

**Keywords:** pollen, grass pollen, aerobiology, climate change, allergy, subtropical, southern hemisphere

## Abstract

Grass pollen is the major outdoor trigger of allergic respiratory diseases. Climate change is influencing pollen seasonality in Northern Hemisphere temperate regions, but many aspects of the effects on grass pollen remain unclear. Carbon dioxide and temperature rises could increase the distribution of subtropical grasses, however, medium term shifts in grass pollen in subtropical climates have not yet been analysed. This study investigates changes in grass pollen aerobiology in a subtropical city of Brisbane, Australia, between the two available monitoring periods, 1994-1999 and 2016-2020. Potential drivers of pollen change were examined including weather and satellite-derived vegetation indicators. The magnitude of the seasonal pollen index for grass showed almost a three-fold increase for 2016-2020 over 1994-1999. The number and proportion of high and extreme grass pollen days in the recent period increased compared to earlier monitoring. Statistically significant changes were also identified for distributions of CO_2_, satellite-derived seasonal vegetation health indices, and daily maximum temperatures, but not for minimum temperatures, daily rainfall, or seasonal fraction of green groundcover. Quarterly grass pollen levels were correlated with corresponding vegetation health indices, and with green groundcover fraction, suggesting that seasonal-scale plant health was higher in the latter period. The magnitude of grass pollen exposure in the subtropical region of Brisbane has increased markedly in the recent past, posing an increased environmental health threat. This study suggests the need for continuous pollen monitoring to track and respond to the possible effects of climate change on grass pollen loads.

## Introduction

Grass pollen is considered to be one of the major outdoor sources of allergens that cause allergic rhinitis in susceptible people during the spring and summer seasons in Australia (1, 2). There is high frequency of allergic rhinitis (19%) and asthma (11%), posing significant health burden (3) in Australia. Thus, it is important to monitor changes over time in allergenic pollen levels and examine potential drivers of these trends, particularly in the light of predicted changes in climate. Few studies present data from subtropical climate zones in lower latitudes.

A growing body of evidence, mostly generated in the Northern Hemisphere, is documenting the way by which climate change affects aerobiology. Climate change appears to influence plant phenology, intensity of airborne pollen seasons, pollen production, and allergenicity (4). The effect of climate change on grass biomass and pollen production is complicated (5) and may be reflected in the changes in temperature, rainfall, carbon dioxide (CO_2_) and ozone levels, soil moisture content, nitrogen deposition, and even species diversity (6–9). For instance, in a 20-year study, Reich et al. (10) found that under elevated CO_2_ there was an increase in biomass production in C4 grasses but a decrease in C3 grass biomass. Furthermore, in controlled experimental environments, pollen and Phl p5 allergen production were found to increase under increased CO_2_ in Timothy (C3) grass (9).

Brisbane is a coastal city at −27.5 degrees latitude, having a humid subtropical climate with typically dry winter/early spring and a hot, wetter summer/early autumn. Brisbane's grass pollen season differs markedly in length and timing from those of temperate regions in Australia (11); lasts longer (up to 6 months), starts around 1 month later, and typically extends from late spring through to mid-autumn (November-April). The response in pollen production of subtropical grasses to temperature, humidity, and rain may differ from those in the C3 subfamilies (12) which dominate in southern Australia, Europe, and North America.

While large scale time series studies of pollen aerobiology have been made in Europe and the USA, no universal trend in grass pollen aerobiology have been identified (13–16). None of these studies cover a coastal subtropical climate like Brisbane. In Europe, Ziello et al. (17) found no significant increase in grass pollen levels across 97 stations over 10-14 years. Similarly, in 26 stations in the USA, Zhang et al. (18) compared the 1990s and 2000s decadal grass pollen season statistics and found no significant changes in season length, start date, peak value, and total pollen load. Emberlin et al. (19) analysed aerobiology data spanning 1961-1993 at three sites in the UK and found pollen levels reflected land use changes outside the city, but that temperature/rainfall correlations varied. Across Spain, Jato et al. (20) analysing data from four Spanish sites spanning 1993-2009, found a decrease in pollen season length and severity and related this to temperature increases. Recently, Anderegg et al. (21) showed, for aggregation of 60 North American pollen monitoring sites, that pollen seasons were commencing significantly earlier, lasting longer and increasing in magnitude, but when pollen was considered in separate categories; weed, grass or trees, grass pollen showed no significant trend in the annual pollen integral. Whilst there is broad agreement amongst most studies that with climate change pollen levels in general are increasing, it is difficult to definitively link airborne pollen changes with temperature and rainfall changes, and to decouple land use changes from climate change (17, 22–25).

To detect and investigate changes in grass pollen aerobiology in a subtropical site represented by Brisbane, Australia, this study aimed to compare records of grass pollen aerobiology between two available data sets, collected respectively in 1994–1999 (the 1990s period) (26) and in 2016–2020 (the 2010s period). Accompanying changes in weather variables and satellite-derived vegetation indices (VI) and ground cover fractions were examined for correlations with pollen aerobiology to provide evidence for potential influencing factors. As Devadas et al. (27) show, satellite-derived vegetation signals and meteorological parameters seem to have a potential to augment understanding of levels and timing of grass pollen.

## Method

**Figure 1:**
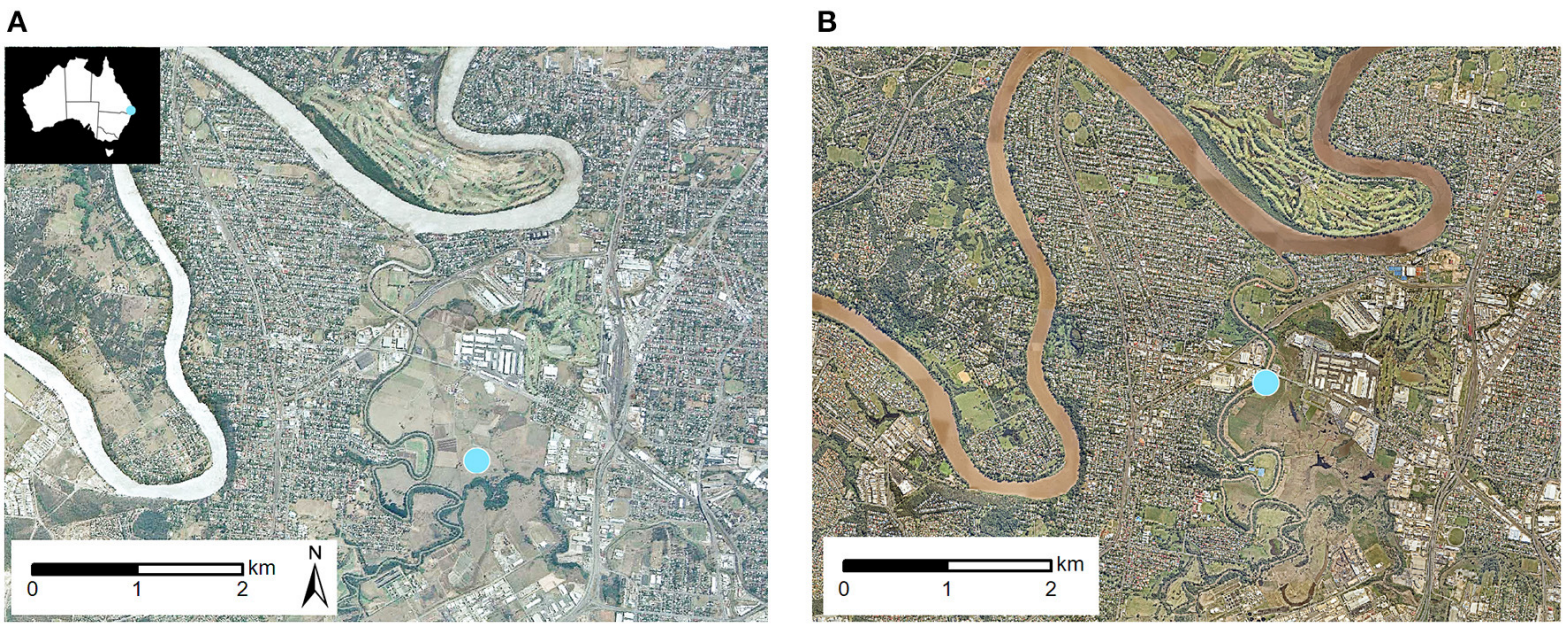
Aerial photographs of Rocklea **(A)** on 4th of October 1994 (QImagery, The State of Queensland 2021) and **(B)** on 11th of April 2017 (Nearmap). Rocklea pollen monitoring site indicated with a Blue marker.

Daily ambient grass pollen data for the 1990s period was obtained from the Green et al. (26) study and pollen data were generated by Queensland University of Technology's Allergy Research Group for the NHMRC AusPollen Partnership Project for the 2010s period (28). These are the only aerobiology datasets available for this site. For both time periods, ambient grass pollen concentrations were monitored at Rocklea, Brisbane, Australia Air Quality Monitoring Station (152.9933, −27.5351) established by the Department of Environment and Science (DES), Queensland. The station was relocated in 2007, however the horizontal difference in location was only around 1 km and both sites were located beside the same series of paddocks (Figure 1). The same instrumentation was used for pollen sample collection: the Burkard 7-day recording volumetric spore traps, Hirst-style sampler (29) (pollen sampler). In the 1990s, the elevation of the pollen sampler was 2 m above ground level and the whole impaction area of the exposed slides was counted to estimate daily grass pollen concentrations. In the 2010s, the height of the pollen sampler was 4 m above ground level and four longitudinal transects of the exposed slides, representing 14% of the impaction area, were counted to estimate the daily grass pollen concentration. Counting methods for both period exceeded minimum best practise requirements articulated in Beggs et al. (30).

A descriptive observational approach was used to compare data between the two monitoring periods. Results were statistically analysed using Spearman correlations between explanatory variables and airborne grass pollen concentrations. As daily pollen concentration distributions were highly skewed, non-parametric tests; Mann-Whitney *U* or Fishers-Exact, were used to compare median or frequency values, respectively.

The following seasonal characteristics adopted from previous studies conducted globally (31–33), were measured in this study: season start date, season end date, length of the pollen season, season magnitude, proportion and severity of grass pollen concentration. The grass pollen season was defined as the number of days after July 1 from the first day to the last day for which grass pollen concentration was above 10 grains/m^3^ of air. SPIn (Seasonal Pollen Integral) was used to investigate changes in magnitude of two grass pollen monitoring periods. SPIn is the sum of all grass pollen concentrations for the season. To estimate the severity of grass pollen exposure, the following grass pollen exposure categories were used; low (0-19 grains/m^3^), moderate (20-49 grains/m^3^), high (50-99 grains/m^3^), and extreme (≥100 grains/m^3^). For comparison with quarterly satellite-derived vegetation metrics, quarterly grass pollen medians were calculated from the daily values.

Linear interpolation was used to fill missing pollen data observations where required (noted as adjusted values). This method was chosen because grass pollen concentration distributions are highly skewed, and the grass pollen concentrations rise and fall across the season; linear fill replaces missing values with values representative of the local temporal stage of the season. Missing data frequency is noted in Supplementary Table 1. The maximum number of contiguous missing days was eight, and (mean, median) for contiguous missing periods were (2.7 days, 1 day) respectively.

Daily meteorological data available for both time periods were obtained from the closest Bureau of Meteorology station at Archerfield, 2 km from the Rocklea site (34): minimum temperature, maximum temperature, rainfall, vapour pressure, solar radiation, mean sea level pressure, and relative humidity at maximum and minimum temperature. Southern hemisphere baseline CO_2_ levels were examined using publicly available BOM/CSIRO data (35).

Landsat Surface Reflectance-Derived Spectral Indices (Normalised Difference Vegetation Index/NDVI and Enhanced Vegetation Index/EVI) and associated quality assurance (QA) layers were downloaded from an online United States Geological Survey (USGS) interface (36). These products have been calculated by the USGS such that they are appropriate for users studying decadal land surface changes across the full history of the Landsat satellite series (37). They are available at ~16-day intervals for the time periods under analysis at a 30 m pixel size. For each available product, clear non-water pixels were identified using bits 1 and 2 of the QA layer, and other pixels discarded. Negative (non-vegetation) NDVI and EVI pixels were also discarded. Distances to the Rocklea pollen sampler were calculated for each pixel and statistics (mean, quartiles) calculated for circular buffers at radii of 5, 10, 25, and 50 km from the sampler, for each 16-day product. To match temporal timing of the available groundcover product (described below), quarterly summaries of the VI data were also produced. To calculate the quarterly mean VI levels for each landcover buffer, the mean was taken of all available 16-day means for each buffer in each quarter. Data products were either missing values or had values of unacceptable quality for ~13% of the possible dates, however there were at least three data sets available for each quarter of each year under analysis.

Changes in groundcover were assessed using the seasonal fractional groundcover product (38) produced by DES. This product is derived by the DES from Landsat satellite images using a well-established and verified algorithm (39). The algorithm extracts information about groundcover specifically, removing information about mid and upper storey vegetation, and hence is suited for herbaceous cover including grasses. For each 30 m square pixel the product provides fractions of green groundcover (photosynthetic herbaceous plants), non-green groundcover (senescent plants, leaf litter), and bare ground. These groundcover data were obtained using VegMachine (40), an online tool accepting a vector area-of-interest and time period and returning groundcover summaries. Using this tool, quarterly data on the fractional components of ground-level vegetation cover (green, non-green, and bare) was extracted for the 27 years (from 1994 to 2020) for circular buffers with radii of 5, 10, 25, and 50 km from the Rocklea pollen monitoring site.

## Results

### Changes in Grass Pollen Data

**Figure 2:**
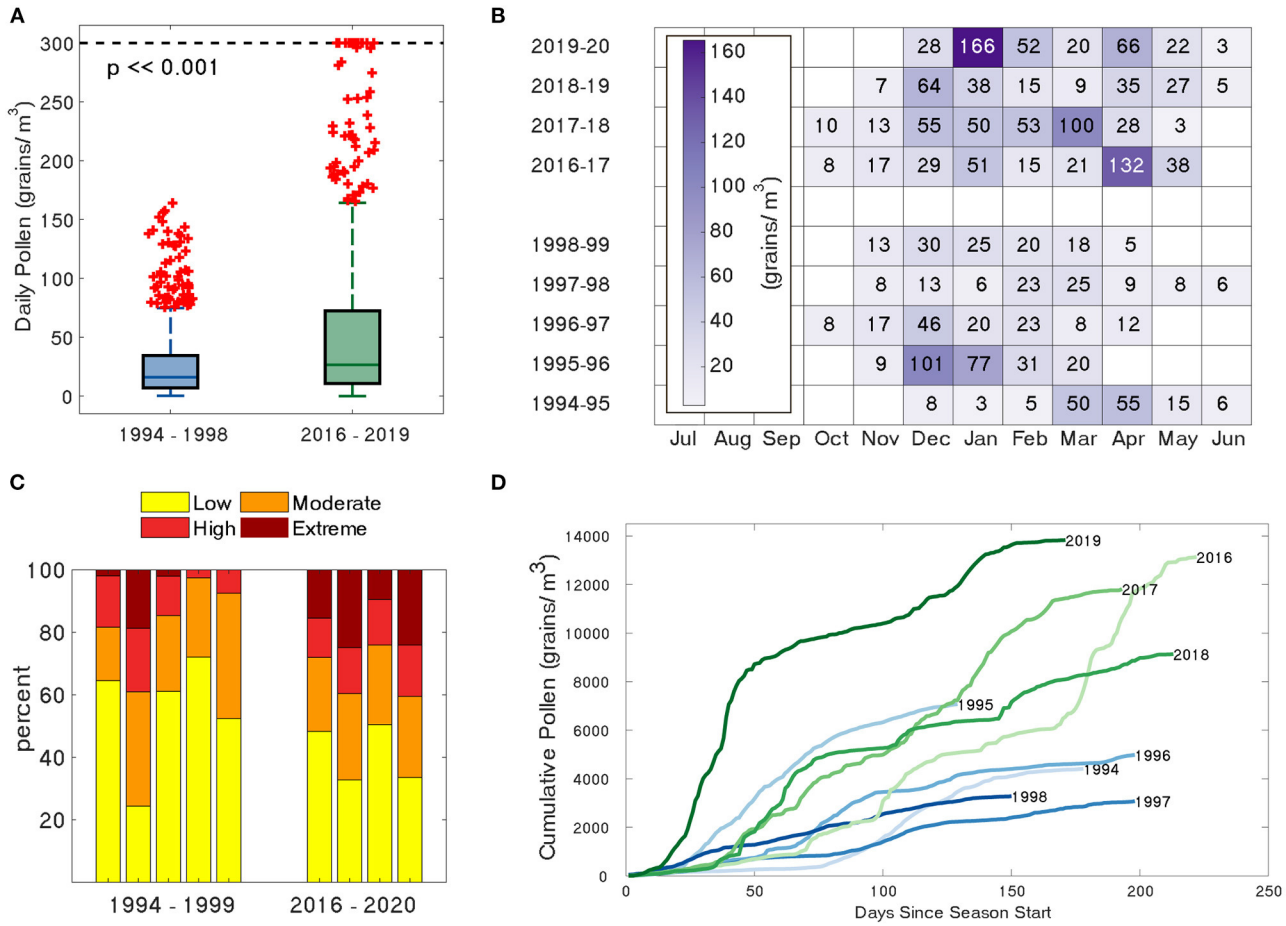
Comparison of **(A)** distribution of daily grass pollen concentrations capped at 300 grains/m^3^ with values greater than three median absolute deviations from the median marked in red. Large proportions of outliers reflect the skewed distribution of the data. **(B)** Distribution of median monthly grass pollen concentration, **(C)** grass pollen severity (proportion of grass pollen in exposure categories) and **(D)** cumulative daily grass pollen concentrations since start of a season, between 1990s (blue) and 2010s (green) periods.

**Figure 3:**
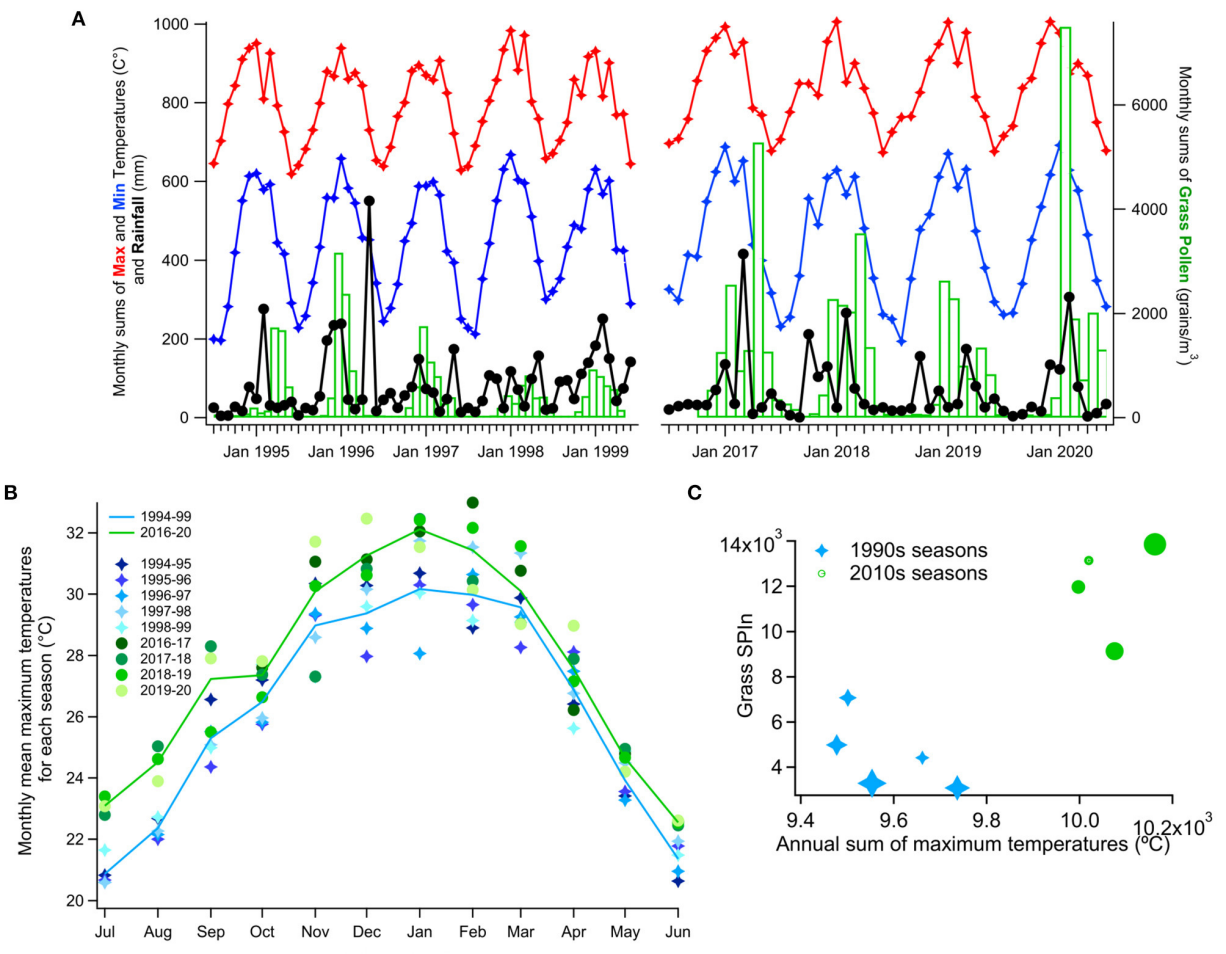
Monthly and annual pollen, rainfall, and temperature. **(A)** Monthly sums of maximum and minimum temperature, rainfall, and grass pollen. **(B)** Monthly mean maximum temperature change between the two monitoring periods. **(C)** Annual pollen and maximum temperature sums (marker's size increases with each season; from 1994-1995 to 1998-1999 and from 2016-2017 to 2019-2020).

Significant differences were found in airborne grass pollen concentrations between the two time periods (Supplementary Table 2). Daily grass pollen concentration distributions were significantly higher during the 2010s than the earlier 1990s period (Figure 2A; Mann-Whitney *U*-test, *p* = 1.7e-17). The median for adjusted SPIns were also higher in the 2010s (10,850; IQR 9,441-12,190) compared to the 1990s monitoring period (3,717; IQR 3,290-5,838) (Supplementary Figure 1; Mann-Whitney *U*-test, *p* = 0.016). The increased pollen intensity of the 2010s data is shown in the distribution of median monthly pollen concentration (Figure 2B), pollen exposure severity categories (Figure 2C), and time-series plots of monthly pollen sums (Figure 3A). The 2010s pollen data had more than twice the proportion of high-to-extreme pollen concentrations recorded in-season (Supplementary Table 3 and Figure 2C; Fishers-Exact test, *p* = 6.5e-26). The highest proportion of high-to-extreme grass pollen were for the 2019, 2017, and 1995 seasons. The pollen seasons of the 2010s had a more complex structure with multiple peaks compared with the 1990s seasons tendency to a single peak (Figures 2B, 3A).

Pollen season timing metrics were not found to differ significantly (Supplementary Table 3). In both data sets the pollen season start dates varied between mid-October and mid-December. Season end dates for the 1990s period were distributed across four calendar months (March-June) and only across two (May-June) for the 2010s. Although the 2010s pollen seasons all finished later in the year, the difference was not statistically significant (Figure 2B; Mann-Whitney *U*-test, *p* = 0.56). Similarly, although the median 2010s season length tended to be greater, the difference between season lengths were not significant (Figures 2B,D; Mann-Whitney *U*-test, *p* = 0.25).

### Changes in Meteorological Variables

Many of the available daily meteorological variables showed significant differences between the two time periods (Supplementary Table 2). Most importantly, daily maximum temperatures during the pollen season were significantly higher in 2010s (Supplementary Figure 2A; Mann-Whitney *U*-test, *p* = 5.4e-06). Monthly mean maximum temperatures (Figure 3B) were significantly and noticeably higher in the 2010s than in the 1990s, particularly in the earlier half of the pollen year. There was also a more pronounced peak of maximum temperature in the middle of the season, around midsummer (January). The SPIns for the 1990s and the 2010s appeared to cluster separately when plotted against the sum of maximum temperatures but this was not significant (Figure 3C).

The difference in daily minimum temperature between periods was not significant (Supplementary Figure 2B). There were also no statistically significant differences found in daily rainfall, or total pollen season rainfall, between the two study periods (also Supplementary Table 4). CO_2_ background levels in the Southern Hemisphere showed an in-season median increase from 360 to 404 ppm, which was significant at a 5% level (*p* = 6e-19) (Supplementary Figure 3).

### Changes in Satellite-Derived Vegetation Indices and Associations With Grass Pollen

**Figure 4:**
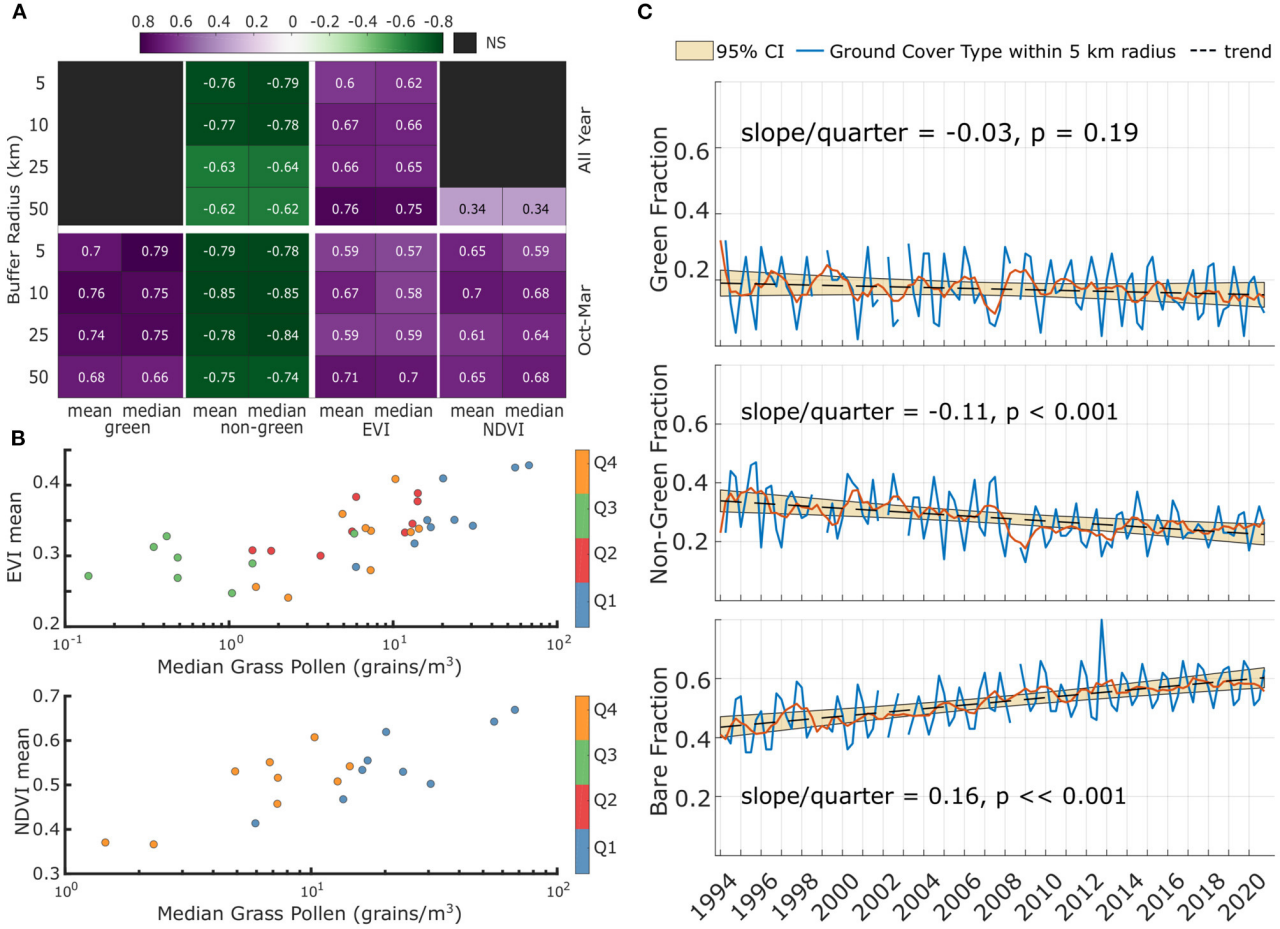
Satellite-derived vegetation measures and correlations with grass pollen within 5 km of the pollen sampler **(A)** Spearman correlations of median quarterly pollen with groundcover fractions (left) and VI (right); **(B)** Scatter plots of mean quarterly VI and median pollen for the 50 km radial buffer; **(C)** Regressions of groundcover fractions over 27 years.

Mean and Median quarterly EVI and NDVI showed significant correlations with median grass pollen (Figure 4A) but differed in how their correlations related to landcover buffer size and quarters of the year. Both VI have increased significantly between the two time periods (Supplementary Figure 4).

Mean and median NDVI at all buffer distances correlated significantly with pollen for the austral spring-summer quarters, but did not show significant correlations when considering all four quarters of the year. (e.g., median NDVI-pollen correlation for the 50 km buffer radius ρ = 0.68, *p* = 0.0025, Figure 4B). NDVI correlations did not change much across the different buffer sizes.

Mean and median EVI correlated significantly with pollen both for the spring-summer quarters and for the whole year's data, but correlations across the full year were strongest. EVI correlations increased with buffer radius with the best correlation between mean EVI and mean pollen for the full years' data within a 50 km radius of the pollen sampler (ρ = 0.76, *p* = 7*e*−07); Figure 4B.

Both NDVI and EVI had significantly higher values in the 2010s compared with the 1990s. For EVI in all quarters a Mann-Whitney *U*-test returned *p* = 0.00107 while for NDVI during the October-March periods a Mann-Whitney *U*-test returned *p* = 0.0259.

### Changes in Ground Cover Fractions and Associations With Grass Pollen

Groundcover changes over the 28-year period are shown in Figure 4C. There was a statistically significant increase in bare land cover fraction (slope = 0.14, *p* < 0.0001) and a decrease in non-green land cover for the 28-year period (fitted slope = −0.08, *p* = 5.6e-04), whereas no medium-term trend was observed in green groundcover fraction over that time period (slope = −0.04, *p* = 0.13).

Significant correlations were found between groundcover fractions, and both mean and median spring and summer quarterly pollen (Figure 4A). For instance, the green groundcover fraction showed significant correlations (ρ = 0.66 to 0.79, *p* = 0.015 to 0.0003) with mean and median quarterly pollen during the October to March quarters of the year, correlating better in the five to 25 km radius around the pollen sampler than for the larger buffer zone. However, the green fraction did not significantly correlate with pollen concentrations when considering all quarters of the year (Figure 4A), for any of the buffer zones analysed, which may reflect intra-annual seasonal fluctuations between herbaceous greening and senescence (Figure 4C).

In contrast there were significant negative correlations of non-green fraction with grass pollen variables for all buffer zones and considering either the whole year or just the October-to-March period. The correlation between pollen and both mean and median non-green fraction was reasonably good (ρ = −0.77 and ρ = −0.78, *p* = 9.8e-7 and 8.4e-8, respectively, for the 10 km buffer).

## Discussion

The distribution of daily in-season grass pollen concentrations and grass pollen SPIns were significantly and markedly higher at the Rocklea monitoring station during the 2010s monitoring period than during the 1990s. It should be noted the pollen season 1995-1996 exhibited concentrations and distribution similar to any year of the 2010s dataset. As well as changes in weather or climate, differences in pollen enumeration procedures, sampler placement, species distribution and land use should be considered as possible explanations for the markedly higher pollen concentrations in the 2010s period.

The same Hirst-style pollen sampler was used in both studies. In the 1990s, the whole of the captured pollen sample was counted whereas in the 2010s the procedure was to use a sample of the daily collection, however both techniques exceed European (41) and Australian standards (30). Addison-Smith et al. (42) showed slightly lower mean values for partial longitudinal tape counting compared with whole-tape counting, which does not help explain the significantly higher values found during 2010s in this study.

A strength of this study is that pollen was collected at two very closely located sites beside the same paddocks (Figure 1). The apparent difference found in monthly pollen distribution over time, from a single major to multiple pollen peaks, may suggest a change in contributing species distribution between monitoring periods. However, our study (8) shows that multiple peaks can also reflect repeated flowering of the same grass species. Certainly, the paddock grass (Rhodes, *Chloris Gayana*) had not changed. Unfortunately though, curation (e.g., harvest frequency, stock load) are undocumented. Skjøth et al. (43) found that local grass pollen sources can be important when measuring urban pollen levels, but also found longer range transport to contribute. We note that extreme pollen levels have been recorded at the Rocklea trap even after grass harvest in the adjoining fields. Detailed mapping of local grass species in Brisbane is unavailable presently but would be extremely useful. The area immediately adjacent to the pollen monitoring site is not expected to be the only contributor to airborne grass pollen levels.

Although changes in grass management proximal to the pollen sampler are unknown, there was no significant change in seasonal fraction of green groundcover within 5 to 25 km of the sampler (Figure 4A and Supplementary Figure 5). This quarterly green groundcover fraction correlated significantly with mean and median quarterly grass pollen concentration for October to March (within the grass pollen season) and up to a 50 km radius zone surrounding the sampler. Seasonal median pollen levels positively correlated with the fraction of green grass-containing land but were negatively correlated with non-green (senescent plant/leaf litter). Statistically significant increases in the seasonal median of satellite-derived plant health indices (EVI and NDVI) were identified between the two monitoring periods indicating a generally higher plant health 2016-2020 than in 1994-1999, which could feasibly lead to greater pollen production in the latter period. It should be noted that some studies (44, 45) report a small positive but surface-dependent bias in NDVI between Landsat 5 and Landsat 7 which is not found in USGS calibration land surfaces. If applicable to land surfaces in our study, bias may be of an order to reduce the statistical significance of the NDVI increase between periods. In this study we have found EVI shows both a more significant increase between study periods and a better correlation with pollen. Notably, the highest correlation of grass pollen concentrations with EVI were in the 50 km radius from the pollen sampler, suggesting that changes in airborne pollen production were not solely dependent on local grass sources or grass land management in the vicinity of the pollen sampler.

Studies have shown that differences in sampler height affect the amount and timing of pollen captured (46–52) but the effect differs between study, sampler height and taxa, and many studies focus on samplers with height differences of 10 to 15 m or greater. In a large data analysis, Rojo et al. (51) found the ratio of daily pollen concentrations (high to low sampler) to range from 0.7 to 2.2. However specifically for samplers with height difference around 2 m like those in our study, very little difference was found between pollen levels of herbaceous plants such as grasses (50). Low level samplers have been shown to be influenced more by local sources than high samplers (51–53). Neither the 1990s nor the 2010s sampler were at ground level, and the 2010s sampler, at 4 m, should be less affected by immediate sources. For this study then, difference in sampler height therefore probably plays less of a role than differences in local sources of grass pollen. It cannot be excluded, however, that change in the site location and/or the pollen sampling height may have influenced the SPIn. However, the fact that PM10 measurements did not change when the Air Quality Monitoring Station moved between sites at Rocklea suggests that the current location is likely similar to the previous site (54).

The significant negative correlation of non-green groundcover with grass pollen needs further examination. If the non-green groundcover within the study region is dominated by senescent grasses, then this could explain the strong inverse relationship, but it also might be coincidental. The long term drop in non-green fraction appears to be balanced by a rise in bare groundcover fraction, most likely due to urbanisation.

Pollen season timing and length did not show significant changes between the two data sets, but this is inconclusive due to uncertainty in the start and end of the 2016 and 2017 seasons, and the end of the 2018 season (missing data), and because the number of pollen seasons was low; five seasons in the 1990s and four seasons in the 2010s. The median green fraction had not changed significantly between periods. No significant change in the fraction of green groundcover coupled with no change in length of pollen season could imply that both the amount of grass in the region and the time over which it produces pollen have not significantly changed. The observed increase in pollen levels might therefore occur from an increased rate of pollen production or increased pollen production per unit ground area, or both (assuming no effect from changes in local grass curation and no difference in airborne pollen transport). Changed rates of pollen production could occur from changes in weather, increases in CO_2_ concentrations (9), species distribution or grass phenology.

Grass pollen concentrations correlated well with the year-round changes in Landsat-derived EVI, and correlate better within a 50 km radius of the pollen sampler than for smaller buffers, particularly during the quarters of the year corresponding to the main grass pollen season. This could be due to a larger fraction of grassland such as pastures found outside the city limits and contributing strongly to the vegetation health signal measured by the EVI. Seasonal and annual EVI distribution during the 2010s period was significantly higher than in the 1990s, implying better median plant health for that time period, and this may contribute to higher pollen production. It should be highlighted that VI were not masked for grasses and hence their observed changes relate to health of all landscape vegetation types and not only grasses. Another contributor to pollen production is CO_2_ level (9). Morgan et al. (7) showed that increases in CO_2_ preferentially supported increase in grass biomass of C4 preferentially over C3 grasses, but that study did not measure pollen production. The significantly higher mean maximum temperature, particularly from July to January observed in the 2010s period, as well as increases in CO_2_ may also have positively influenced pollen production. The rise in CO_2_ observed here aligns with a global trend of increase in CO_2_ (55).

The results of this study emphasise an underlying need for continuous pollen monitoring to track and evaluate effects related to climate changes. Importantly, our site in Rocklea is only one example of a subtropical pollen monitoring site. There are relatively few pollen monitoring sites located in subtropical zones (56). Further long-term pollen monitoring in other subtropical climate zones should be supported and examined. Moreover, it should be highlighted that there are only few long-term pollen monitoring sites in the Southern Hemisphere with which to generate important knowledge of trends in aerobiology and assess climate change impacts on pollen exposure (5).

Whilst several time series aerobiological monitoring studies have shown effects on the timing of pollen seasons in Northern Hemisphere including Europe (17) and the US (21), no universal trend in grass pollen aerobiology has been found. Few studies have discovered significant changes in grass pollen aerobiology (16), but none has associated changes in grass pollen seasonal index with observable meteorological factors attributed to climate change (13–15). This study, using data from a subtropical climate zone rather than aggregating data from multiple sites spanning climate zones, has found a significant increase in both daily pollen concentrations and grass pollen SPIn between two periods of monitoring in the 1990s and 2010s, without significant change in green groundcover fraction over the 28-year period. These pollen changes were positively correlated with increased maximum median monthly temperature, and coincided with significant increases in atmospheric CO_2_, and increases in satellite-derived seasonal vegetation health indices, although a relationship with rainfall was not evident. However, factors other than climate change, including changes in land cover and land management, changes in species compositions or ratios of C3 and C4 grass populations (8), may also be driving the observed medium-term differences in grass pollen aerobiology in Brisbane.

The outcomes of this research contribute new insights to prompt better environmental public health surveillance systems for preventive management of allergic respiratory diseases in Australia. Given that subtropical climate zones are widening, and the geographical distribution of subtropical species are expanding away from the equator, understanding the effect of climate change on plant diversity and aerobiology of subtropical grasses will become more relevant globally.

## Data Availability Statement

Publicly available datasets were analyzed in this study. This data can be found here: https://data.aurin.org.au/dataset/tern-tern-rocklea-pollen-count-weekly-by-pollen-type-1994-99-na http://www.bom.gov.au/climate/data/?ref=ftr. https://www.csiro.au/en/research/natural-environment/atmosphere/latest-greenhouse-gas-data https://espa.cr.usgs.gov/ https://vegmachine.net/ https://nextcloud.aurin.org.au.

## Author Contributions

BA-S, AM, DD, and JD contributed to conception and design of the study and drafted the manuscript. BA-S, AM, and DD performed statistical analysis. All authors contributed to data collection, manuscript revision, and read and approved the submitted version.

## Conflict of Interest

JD reports financial co-contribution from Stallergenes Greer Australia Pty Ltd., as well as in kind non-financial support from Stallergenes Greer Australia Pty Ltd. during the conduct of the study. JD reports grants from Abionic SA outside the submitted work. In addition, JD reports QUT has patents broadly relevant US PTO 14/311944 and AU2008/316301 issued. The remaining authors declare that the research was conducted in the absence of any commercial or financial relationships that could be construed as a potential conflict of interest.

## Publisher's Note

All claims expressed in this article are solely those of the authors and do not necessarily represent those of their affiliated organizations, or those of the publisher, the editors and the reviewers. Any product that may be evaluated in this article, or claim that may be made by its manufacturer, is not guaranteed or endorsed by the publisher.
