# Supplementary material for: Medium-Term Increases in Ambient Grass Pollen Between 1994-1999 and 2016-2020 in a Subtropical Climate Zone
Source: Front Allergy. 2021 Aug 5;2:705313. doi: 10.3389/falgy.2021.705313 (PMC8974679; doi:10.3389/falgy.2021.705313)
Supplement: Supplementary file 1 [file Data_Sheet_1.PDF]

Supplementary Material

Medium-term increases in ambient grass pollen between 1994-1999 and 2016-2020 in a subtropical climate zone

Beth Addison-Smith[1†], Andelija Milic[1†], Divya Dwarakanath[1†], Marko Simunovic[1], Shanice Van Haeften[1], Victoria Timbrell[1], Janet M. Davies[1,2*]

[1]School of Biomedical Sciences, Centre for Immunology and Infection Control, and Centre for the Environment, Queensland University of Technology, Brisbane, QLD, Australia

[2]Office of Research, Metro North Hospital and Health Service, Brisbane, QLD, Australia

†These authors share first authorship.

***Correspondence:**
Janet M. Davies
j36.davies@qut.edu.edu

1 Supplementary Figures and Tables

1.1 Supplementary Figures

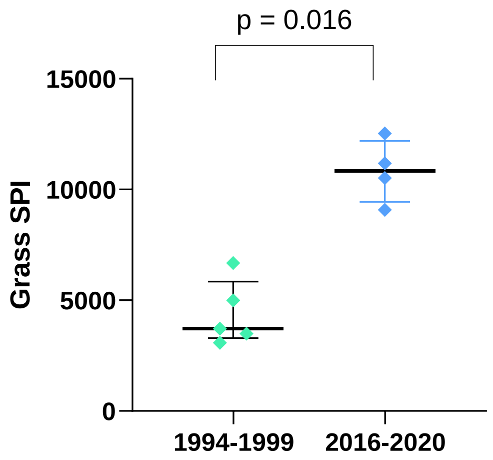

Supplementary Figure 1. Total adjusted Seasonal Pollen Integral (SPI)

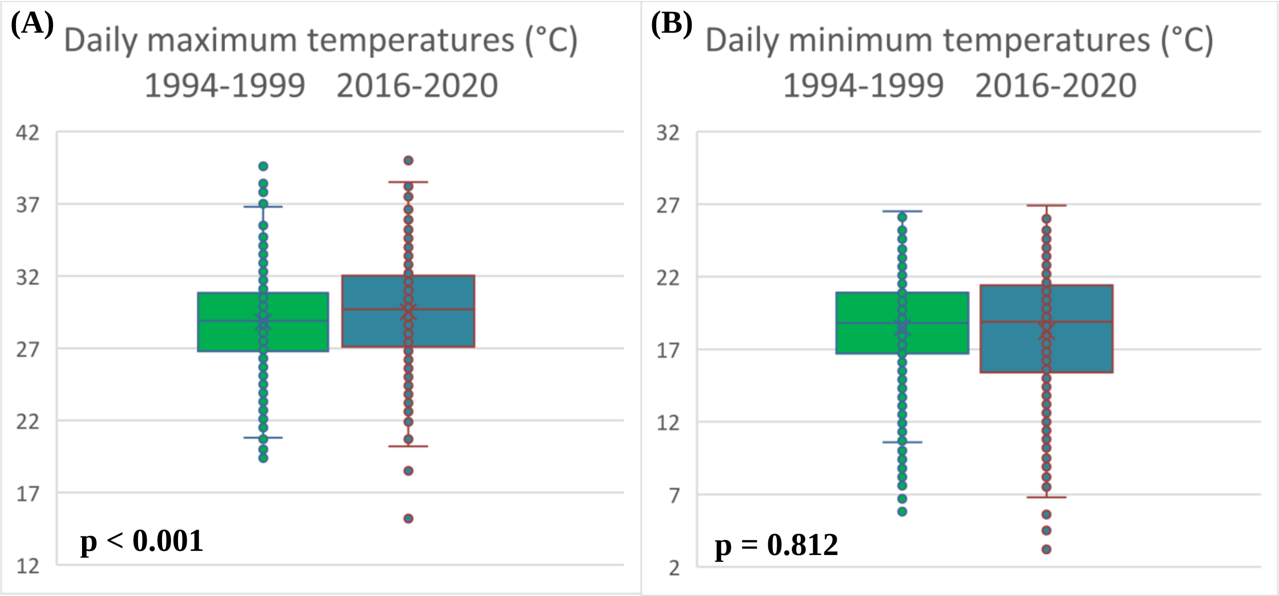

Supplementary Figure 2. Changes in daily maximum and minimum temperatures between periods

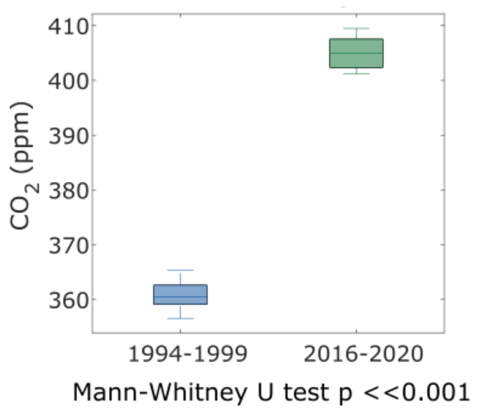

Supplementary Figure 3. Change in monthly atmospheric CO_2 levels between the two monitoring periods

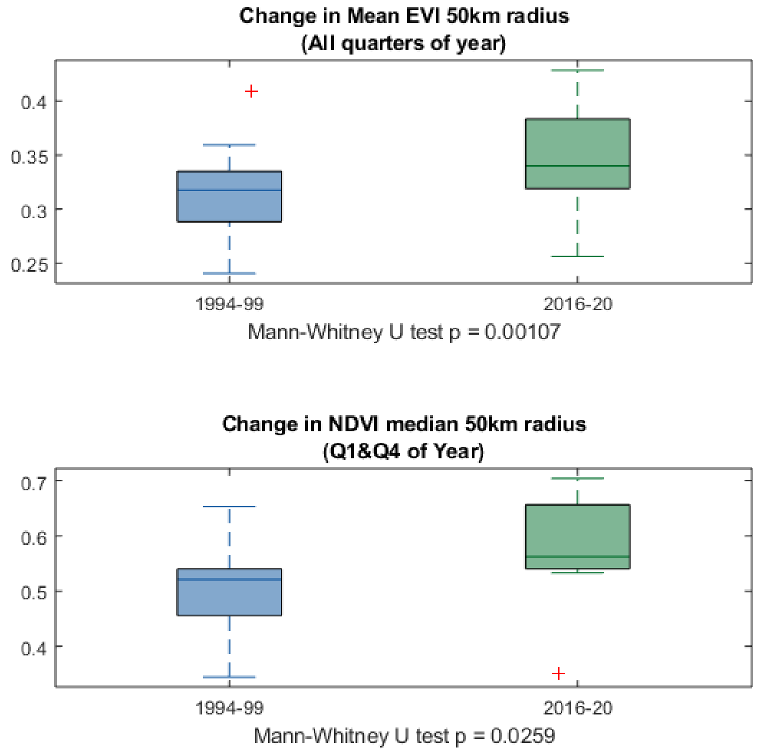

Supplementary Figure 4. Changes in mean quarterly EVI and NDVI values between the two periods

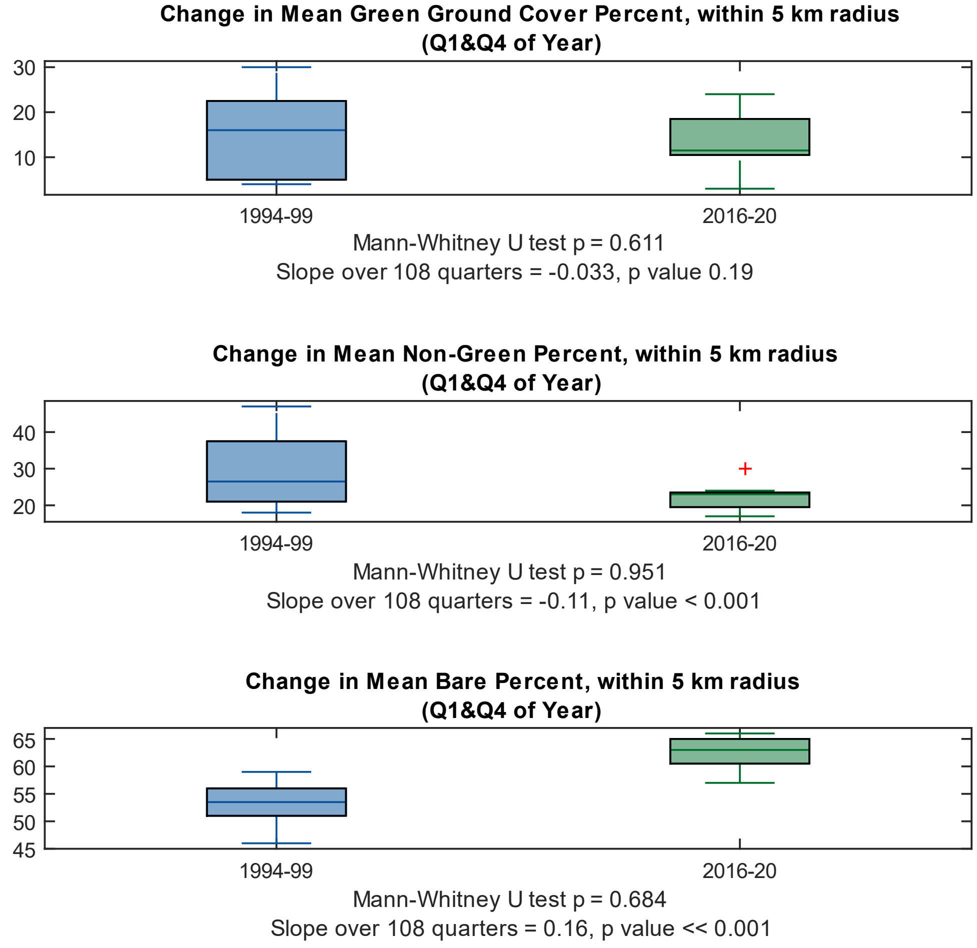

Supplementary Figure 5. Changes in quarterly mean groundcover fraction (Q1 and Q4) between the two periods, and indicating regression results for data from all available quarters 1994-2020.

1.2 Supplementary Tables

Supplementary Table 1. Missing data

Grass pollen season	Number of missing data and days in season	Days in contiguous missing day clusters
1994-1995	20 out of 178	[7,7,1,5]
1995-1996	6 out of 129	[6]
1996-1997	0 out of 198	[]
1997-1998	1 out of 198	[1]
1998-1999	3 out of 150	[1,1,1]
2016-2017	15 out of 222	[3,1,1,7,3]
2017-2018	16 out of 193	[1,1,1,1,1,1,2,7,1]
2018-2019	1 out of 213	[1]
2019-2020	13 out of 171	[1,3,8,1]

Supplementary Table 2. Mann-Whitney U test results

Variable	P value	Change	Quanta
		**Weather**	
**daily grass pollen**	1.7E-17	new > old	Daily
**daily_rain**	0.167	NS	Daily
**max_temp**	5.4E-06	new > old	Daily
**min_temp**	0.812	NS	Daily

**vp**	3.8E-07	new < old	Daily
**radiation**	5.6E-05	new < old	Daily
**rh_tmax**	1.8E-26	new < old	Daily
**rh_tmin**	1.8E-15	new < old	Daily
**mslp**	7.8E-03	new > old	Daily
**CO_2**	5.58e-19	new > old	Monthly
**Satellite-derived Indices**			
**EVI mean (50km buffer)**	.00107	new > old	Quarterly, all quarters
**NDVI median (50km buffer)**	.0259	new > old	Quarterly, winter quarters
**green fraction**	.611	NS	Quarterly, winter quarters
**non-green fraction**	.951	NS	Quarterly, winter quarters
**bare fraction**	.684	NS	Quarterly, winter quarters

Supplementary Table 3. Pollen Summary data

pollen season	start date	end date	days	SPI	Median	IQR	mean	std	percent high-extreme
**1994-1995**	15/12/94	10/6/95	178	4416	9.6	38.6	24.8	27.5	18%
**1995-1996**	10/11/95	17/3/96	129	7073	41.7	60.9	54.8	42.6	39%
**1996-1997**	12/10/96	27/4/97	198	4985	13.8	24.0	25.2	27.8	15%
**1997-1998**	20/11/97	5/6/98	198	3081	11.9	15.4	15.6	12.7	3%
**1998-1999**	14/11/98	12/4/99	150	3285	19.5	21.0	21.9	14.6	7%
**2016-2017**	9/10/16	18/5/17	222	13136	21.9	58.3	59.2	86.1	28%
**2017-2018**	29/10/17	9/5/18	193	11965	38.4	89.2	62.0	60.3	40%
**2018-2019**	3/11/18	3/6/19	213	9137	19.9	39.9	42.9	62.8	24%
**2019-2020**	20/12/19	7/6/20	171	13851	35.9	95.9	81.0	112.8	41%

Supplementary Table 4. Monthly sums of Rainfall (mm) for each season (colour gradient applied; more intense the colour is, higher the rainfall sum value).

	Jul	***Aug***	***Sept***	***Oct***	***Nov***	***Dec***	***Jan***	***Feb***	***Mar***	***Apr***	***May***	***Jun***
1994-95	25.3	4.4	5	27.4	16.7	78	47.5	276.3	30.8	24.9	31.3	40
1995-96	5.8	24.2	19	53.8	196.3	235	238.8	45.8	21.4	45.7	550.6	16.8
1996-97	45.2	62.1	25	56.2	77.8	148.6	72.8	63.4	15	47.4	174.2	14
1997-98	24.6	14.6	42.2	107.2	98.6	24	117.4	71	28.8	98.5	157	20.2
1998-99	23.6	91.2	94.6	47.2	111	139.6	183	251.6	150.2	42.2	74.2	141.7
2016-17	20.6	29	33.8	32.2	31.8	70.4	135.4	35	416.2	9.4	26	60.8
2017-18	30.2	6	0.6	212	103.8	129.6	25.6	266.4	74.2	34	19.6	26
2018-19	17.8	17.8	23.6	155.8	22.6	68.2	26.2	34.4	174.6	79.6	26.2	47.8
2019-20	16.8	3.6	8.4	27	16	134.4	122.7	306.8	78.4	3.2	11.4	34.4
